# P-1620. Use of Multiplex PCR and Antimicrobial Stewardship Intervention to Optimize Antimicrobial Therapy in Pneumonia Patients at a Community Teaching Hospital

**DOI:** 10.1093/ofid/ofae631.1787

**Published:** 2025-01-29

**Authors:** Paula A Politis, Matthew England, Thomas M File

**Affiliations:** Summa Health System, Akron, Ohio; Summa Health, Akron, Ohio; Summa Health System, Akron, Ohio

## Abstract

**Background:**

Patients admitted to the hospital with community-acquired pneumonia (CAP) or those who develop hospital-acquired pneumonia (HAP) are usually treated with broad spectrum antimicrobials for several days while awaiting the results of gold standard respiratory culture. Recently, our health system instituted a rapid Pneumonia PCR Panel (PN) that has increased the yield of pathogen-specific, actionable results in significantly less time than culture allowing for faster optimization of antimicrobial therapy. Our Antimicrobial Stewardship Program (ASP) routinely follows and provides recommendations based on the results of the PNs. The purpose of our quality improvement project was to assess the optimization of antimicrobial therapy associated with use of the PNs and ASP intervention.

Table 1

**Figure:**
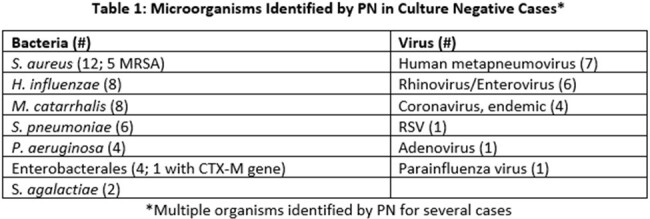

**Methods:**

We performed a retrospective review of patients admitted to our institution on whom ASP intervened with a PN performed for the timeframe of January 2023-March 2023. We assessed ASP intervention type, respiratory culture and PN results, and patient location.

**Results:**

There was a total of 91 patients with PNs/respiratory cultures evaluated. 52 (57%) patients were in the ICU. 38% of cultures were positive with > 1 bacterial organism isolated whereas 82% of PNs were positive with > 1 organism identified. 34/35 of positive cultures had the same organism identified on PN. Of negative cultures (no growth, flora, or *C. albicans*), 42/56 had a positive PN for a potential pathogen, including viruses and/or bacteria. Microorganisms identified by PN in culture negative cases are listed in Table 1. In 73% of patients, the PN lead to recommendations by the ASP for more appropriate antimicrobials. Interventions included: de-escalation (50), change antibiotic for better coverage (21), duration of therapy (32), discontinue antibiotic (3), other (5), and recommend initial PN (9). In cases where ASP recommended the PN, 6 patients were appropriately covered or de-escalated by the medical team, 2 had ASP intervention, and 1 patient expired.

**Conclusion:**

The use of the PN increased the yield of potential etiologic pathogens in patients with CAP and HAP, and when paired with ASP recommendations led to overall optimized antibiotic use and enhanced antimicrobial stewardship.

**Disclosures:**

**Thomas M. File, Jr., MD, MSc, MACP, FIDSA**, Eagle Pharmaceuticals: Member of DSMB for clinical trial|MicroGenDx: Advisor/Consultant|Nabriva: Advisor/Consultant|Paratek Pharmaceuticals: Advisor/Consultant|Pfizeer: Grant/Research Support|shionogi: Advisor/Consultant|thermoFisher Scientific: Advisor/Consultant

